# A neural model of the optomotor system accounts for ordered responses to decreasing stimulus spatial frequencies

**DOI:** 10.1186/1471-2202-16-S1-P159

**Published:** 2015-12-18

**Authors:** Alex Cope, Chelsea Sabo, Eleni Vasilaki, Kevin Gurney, James AR Marshall

**Affiliations:** 1 Department of Computer Science, University of Sheffield, Sheffield, S10 2TN, UK; 2 Department of Psychology, University of Sheffield, Sheffield, S10 2TN, UK

## 

**Figure 1:**
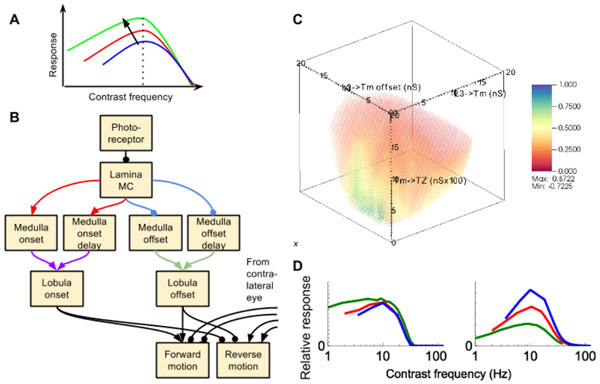
**A. Cartoon of ordering effect indicated by honeybee descending neuron responses**. Spatial frequency decreases from blue to green. **B**. Model diagram showing annealed synapses (coloured, same colours indicate same synaptic conductance). **C**. Slice of annealing results (Medulla-Lobula conductance of 0.04nS) showing stable region (green / blue area). **D**. Model response for a high objective function value (left), and the corresponding response for an RHD using the model output up to the Medulla.

In insects the optomotor response produces a motor action to compensate for unintended body rotation. The response is generally modeled as a Reichardt-Hassenstein (HSD) or Barlow-Levick (BL) correlation detector, as anatomical and physiological studies in Drosophila melanogaster have demonstrated consistent neural pathways and responses in the insect brain [1]. Recordings from the descending neurons carrying the optomotor response signal in honeybees indicate an ordering effect for different stimulus spatial frequencies, with a greater response with decreasing frequency [2] (see Figure 1A), which is not accounted for by HSD or BL correlation detectors.

We present a model in the SpineML format of the optomotor system, using Izhikevich point neurons tuned to match the respective physiological responses, which is shown in Figure 1B. To examine if the model reproduces the ordering effect found in the honeybee we performed simulated annealing on four conductance values in the model, as shown in Figure 1. The objective function is designed to maximize: correct ordering; a 10Hz maximum response; and contrast between responses to forward and reverse motion. **A**. The data was imported into a commercial software package (MATLAB 7.14, The MathWorks Inc., Natick, MA, 2012) for analysis and interpolated onto a 41^4 ^grid. A 3D slice of this 4D grid can be seen in Figure 1C. Spatial frequencies of 32.7, 18.9 and 9.5 Hz are used.

A stable region in which a high value of the objective function, and thus correct spatial frequency ordering, could be obtained was found. In the stable region the onset pathway activity is low, leading to offset activity dominating. A RHD using the model up to the Medulla does not show correct ordering.
